# Supplementary material for: Experiences of frontline healthcare workers and their views about support during COVID-19 and previous pandemics: a systematic review and qualitative meta-synthesis
Source: BMC Health Serv Res. 2021 Sep 6;21:923. doi: 10.1186/s12913-021-06917-z (PMC8419805; doi:10.1186/s12913-021-06917-z)
Supplement: Supplementary file 1 — Additional file 1. [file 12913_2021_6917_MOESM1_ESM.docx]

**Appendix 1. CASP results for individual studies**

|  | **1. Was there a clear statement of the aims of the research?** | **2. Is a qualitative methodology appropriate?** | **3. Was the research design appropriate to address the aims of the research?** | **4. Was the recruitment strategy appropriate to the aims of the research?** | **5. Were the data collected in a way that addressed the research issue?** | **6. Has the relationship between researcher and participants been adequately considered?** | **7. Have ethical issues been taken into consideration?** | **8. Was the data analysis sufficiently rigorous?** | **9. Is there a clear statement of findings?** | **10. How valuable is the research?** |
| --- | --- | --- | --- | --- | --- | --- | --- | --- | --- | --- |
| Aghaizu et al. (2011) | T | T | T | T | T | N | T | T | T | T |
| Al Knawy et al. (2019) | T | T | T | P | T | P | N | T | T | T |
| Andertun et al. (2016) | T | T | T | T | T | P | T | T | T | T |
| Bensimon et al. (2007) | T | T | T | T | P | T | P | T | T | T |
| Bergeron et al. (2006) | T | T | P | P | P | N | P | P | T | T |
| Broom et al., (2017) | T | T | T | P | N | N | P | P | T | T |
| Chen et al. (2020) | N | T | P | N | P | N | N | N | T | T |
| Chiang et al. (2007) | T | T | T | P | T | N | T | T | P | P |
| Chung et al. (2004) | T | T | P | T | T | T | T | T | T | T |
| Erland & Dahl (2017) | T | T | T | T | T | T | T | T | T | T |
| Fawaz & Samaha (2020) | T | T | T | T | T | T | T | T | T | T |
| Gershon et al. (2016) | T | T | T | P | T | P | T | T | T | T |
| Guimard et al. (1999) | P | T | T | N | P | N | N | N | P | P |
| Hewlett & Hewlett (2005) | T | T | T | P | P | N | N | N | T | P |
| Honey & Wang (2012) | T | P | T | T | T | N | P | P | T | T |
| Im et al. (2018) | T | T | T | P | P | N | N | N | P | P |
| Ives et al. (2009) | T | T | T | T | T | N | P | T | T | T |
| Kim (2018) | T | T | T | T | T | P | T | T | T | T |
| Koh et al. (2011) | T | T | T | T | T | P | N | T | T | T |
| Lam & Hung (2012) | T | T | T | T | T | T | T | T | T | T |
| Lamb (2018) | T | T | T | T | T | T | T | P | P | T |
| Lau & Chen (2004) | T | T | T | N | P | N | N | P | P | T |
| Lee et al. (2005) | T | T | T | T | P | N | P | T | T | T |
| Liu & Liehr (2009) | T | T | T | T | T | N | P | T | T | P |
| Liu et al. (2019) | T | T | T | T | T | P | T | T | T | T |
| Liu et al. (2020) | T | T | T | T | T | T | T | T | T | T |
| Locsin et al. (2009) | T | T | T | T | T | P | P | P | T | T |
| McMahon et al. (2017) | T | T | T | T | T | P | T | T | T | T |
| Mok et al. (2005) | T | T | T | P | P | N | T | T | T | P |
| Moore et al. (2005) | T | T | T | P | P | N | N | P | P | P |
| O’Boyle et al. (2006) | T | T | T | T | T | N | T | T | T | T |
| O'Sullivan et al. (2009) | T | T | T | T | T | N | N | T | T | T |
| Pearce et al. (2011) | T | T | T | T | P | N | N | N | T | T |
| Raven et al. (2018) | T | T | T | N | T | T | T | T | T | T |
| Raven, Wurie, & Witter (2018) | T | T | T | T | T | N | T | T | T | T |
| Rubin et al. (2016) | T | T | T | P | P | T | T | T | T | T |
| Sarikaya & Erbaydar (2007) | T | T | T | P | P | N | P | T | P | T |
| Shaw et al. (2006) | T | T | T | T | T | P | P | P | T | T |
| Shih et al. (2007) | T | T | T | P | T | T | T | T | T | P |
| Shih et al. (2008) | T | T | T | P | T | P | T | T | T | T |
| Smith et al. (2017) | T | T | T | P | P | N | P | P | T | P |
| Sun et al. (2020) | T | T | T | T | T | T | T | T | T | P |
| Taylor et al. (2018) | T | T | T | P | T | N | P | T | T | T |
| von Strauss et al. (2017) | T | T | T | P | P | N | T | P | T | T |
| Wong et al. (2011) | T | T | T | P | P | N | T | T | T | T |
| Yin & Zeng (2020) | T | T | T | P | P | N | T | T | T | T |

**T = totally met, P = partially met, N = not met**

**Appendix 2. Search Strategy**

**PsychINFO search strategy**


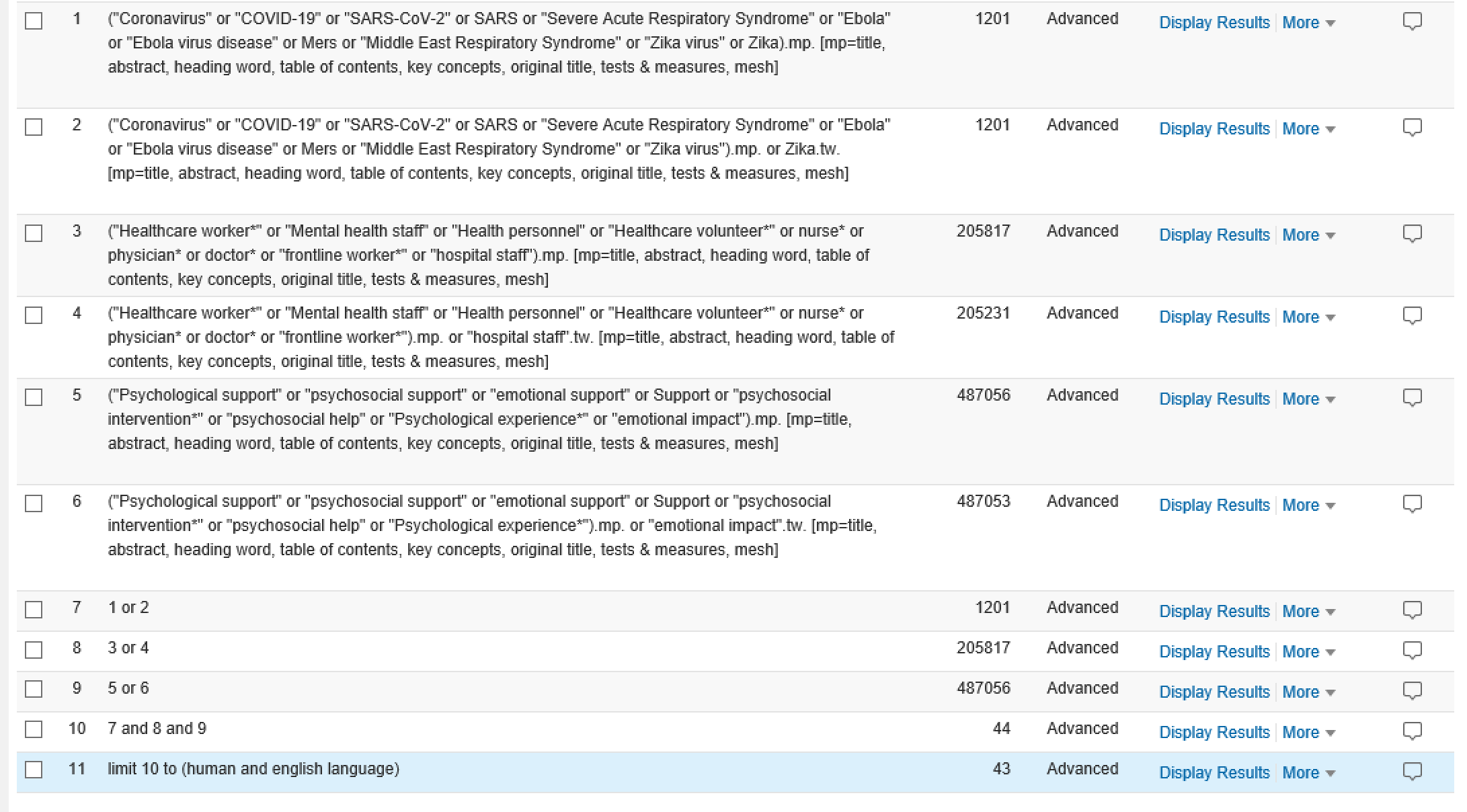


**Medline search strategy**


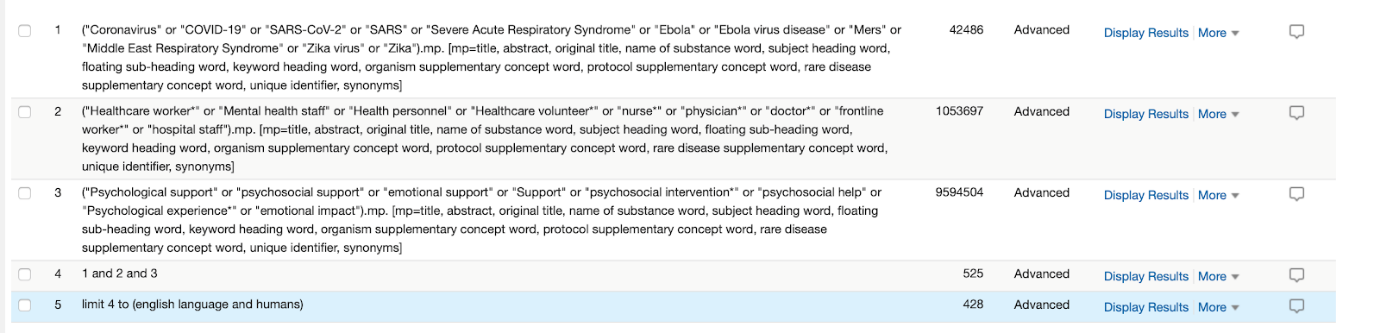


**PubMed search strategy**

“Coronavirus” OR “COVID-19” OR “SARS-CoV-2” OR SARS OR “Severe Acute Respiratory Syndrome” OR “Ebola” OR “Ebola virus disease” OR “Mers” OR “Middle East Respiratory Syndrome” OR “Zika virus” OR Zika

AND

“Healthcare worker*” OR “Mental health staff” OR “Health personnel” OR “Healthcare volunteer*” OR nurse* OR physician* OR doctor* OR frontline worker* OR “hospital staff”

AND

“Psychological support” OR “psychosocial support” OR “emotional support” OR Support OR “psychosocial intervention*” OR “psychosocial help” OR “Psychological experience*” OR “emotional impact”
